# Antifungal Activity of *Aniba canelilla* (Kunth) Mez Essential Oil and Its Main Compound 1-Nitro-2-Phenylethane against Dermatophytes

**DOI:** 10.3390/antibiotics13060488

**Published:** 2024-05-24

**Authors:** Tainá Kreutz, Eliane Oliveira Salines Duarte, Priscilla Maciel Quatrin, Simone Braga Carneiro, Valdir F. Veiga-Junior, Alexandre Meneghello Fuentefria, Letícia S. Koester

**Affiliations:** 1 Programa de Pós-Graduação em Ciências Farmacêuticas, Faculdade de Farmácia, Universidade Federal do Rio Grande do Sul, Av. Ipiranga, Santana, 2752, Porto Alegre 90610-000, Rio Grande do Sul, Brazil; tainakreutz@gmail.com (T.K.); alexandre.fuentefria@ufrgs.br (A.M.F.); 2 Faculdade de Farmácia, Universidade Federal do Rio Grande do Sul, Av. Ipiranga, Santana, 2752, Porto Alegre 90610-000, Rio Grande do Sul, Brazil; elianesalines@gmail.com; 3 Programa de Pós-Graduação em Microbiologia Agrícola e do Ambiente, Faculdade de Farmácia, Universidade Federal do Rio Grande do Sul, Av. Ipiranga, Santana, 2752, Porto Alegre 90610-000, Rio Grande do Sul, Brazil; 4 Departamento de Química, Instituto de Ciências Exatas, Universidade Federal do Amazonas, Av. Gal. Rodrigo Octávio, Japiim, 6200, Manaus 69079-000, Amazonas, Brazil; braga.simone.c@gmail.com (S.B.C.); valdir.veiga@gmail.com (V.F.V.-J.); 5 Programa de Pós-Graduação em Química, Instituto Militar de Engenharia, Praça General Tibúrcio, Urca, 80, Rio de Janeiro 22290-270, Rio de Janeiro, Brazil

**Keywords:** *Aniba canelilla*, 1-nitro-2-phenylethane, antifungal activity, dermatophytes, minimum inhibitory concentration

## Abstract

The essential oil of *Aniba canelilla* (Kunth) Mez (EOAC), an Amazon plant composed of a rare nitro compound, has shown scientific evidence of antifungal activity but is still unexplored against dermatophytes. The antifungal susceptibility of EOAC and its main compound, 1-nitro-2-phenylethane (NP), was evaluated against dermatophytes (*Trichophyton rubrum*, *T. mentagrophytes* and *Microsporum canis*), evidencing antifungal activity with an inhibitory concentration lower than 256 μg/mL. The mechanism of action was also evaluated, and it is suggested that EOAC and NP have fungicidal action in the fungal membrane, since the antifungal activity occurs through a modification of the shape of the conidial structures of the fungus, showing the permeability of the intracellular content due to the visually observed plasmolysis and cytosolic extravasation through an osmotic process. These results suggest the essential oil and its main compound are promising plant-derived alternatives for treating ungual dermatophytosis.

## 1. Introduction

Onychomycosis is a fungal infection which represents almost 50% of all nail disorders, causing local pain, nail discoloration and social inconvenience [1,2]. Although onychomycosis has fungi of different genera as its infectious agents, dermatophytes still prevail in most cases, with *Trichophyton rubrum* and *Trichophyton mentagrophytes* being liable for 60–70% of infections, especially affecting individuals with greater immunological susceptibility to fungal opportunism, such as diabetics or even patients undergoing immunosuppressive treatment [3,4]. The treatment of onychomycosis consists of oral and/or topical treatments [5,6]. However, due to the increasing reports of fungal resistance to conventional treatments, there is a compelling need for new pharmaceuticals and therapeutic proposals to overcome nail infections [6,7,8].

*Aniba canelilla* (Kunth) Mez belongs to the Lauraceae family, known for its several aromatic species of economic importance. *A. canelilla* is a famous Amazonian species that is known for its aromatic bark that resembles cinnamon (*Cinnamomum zeylanicum* L.), being popularly known as “precious bark”. Rich in benzenoids, it also presents cinnamaldehyde and benzaldehyde, but its major compounds are 1-nitro-2-phenylethane (NP), a rare plant nitro compound, and methyleugenol (ME), a eugenol-derived phenylpropanoid; together, these make up about 90–99% of the volatile essential oil [9,10]. *A. canelilla* essential oil (EOAC) has been studied against dermatitis, since several antifungal properties against *Candida* spp. and phytopathogens have been observed [11,12,13,14,15]. Other studies have also confirmed trypanocide and leishmanicide effects [16,17]. Natural nitro compounds are rare in nature, especially in plants. To the best of our knowledge, the processes of detecting and isolating 1-nitro-2-phenylethane have only been described for *A. canelilla*. Antifungal activities against clinical isolates of *Candida* spp. and *Aspergilus fumigatus* have already been reported. In experiments performed in broth microdilution, the minimum inhibitory concentration (MIC) was around 170 μg/mL, 360 μg/mL and 720 μg/mL for *Candida albicans*, *C. parapsilosis* and *C. tropicalis*, respectively. In a clinical isolate of *Aspergilus fumigatus*, the (MIC) was around 1500 μg/mL. These results are promising considering the high toxicity of this compound against yeasts, especially against *C. albicans* [18]. Despite these scientific indications of antifungal activity, there is no report so far on the activity of *A. canelilla* essential oil and its main compound, 1-nitro-2-phenylethane, against dermatophytes. Additionally, there is no information on their use as an alternative treatment for fungal resistance in dermatophytosis and onychomycosis.

The topical treatment of onychomycosis faces challenges due to the barrier character of the nail plate, which leads to poor drug permeation and consequently low drug concentrations in deeper layers of the nail. To expand the use of topical antifungal therapy in onychomycosis, there is a need for new compounds and technological strategies to enhance the permeation of drugs through the nail plate and achieve efficient concentrations at infection sites. In a recent study, our research group has demonstrated the ability of NP and ME to permeate to a great extent through porcine hooves placed between donor and receptor chambers of Franz diffusion cells. A 150 μL blend of EOAC in mineral oil (10%, *v*/*v*) containing Span 80 (2.5%, *v*/*v*) was placed in contact for 72 h with the standardized hoof membrane. The amount of NP and ME retained in the porcine hoof membrane was 1272.6 ± 225.7 μg/cm^2^ and 84.7 ± 20.4 μg/cm^2^, respectively. Although ME could not be detected in the receptor fluid, NP reached this compartment in the order of 441.1 ± 92.1 μg/cm^2^. This interesting result indicates that NP has the potential to completely cross all nail plate layers and reach the nail bed, which is the site of infection of some subtypes of this disease [19]. Thus, its permeation properties allow NP to be studied in several other biological models where the difficult task of reaching the infection target is a necessity.

Supported by an unusual bioactive substance with distinguished permeation properties, this research focused on evaluating the biological potential of *A. canelilla* essential oil (EOAC) and its main compound (1-nitro-2-phenylethane-NP) through microbiological assays against clinical isolates of the dermatophytes *Trichophyton rubrum*, *Trichophyton mentagrophytes* and *Microsporum canis*. Their mechanisms of action were also evaluated.

## 2. Results

### 2.1. Chemical Composition of Aniba canelilla Essential Oil (EOAC) and Its Main Compound NP

**Figure 1:**
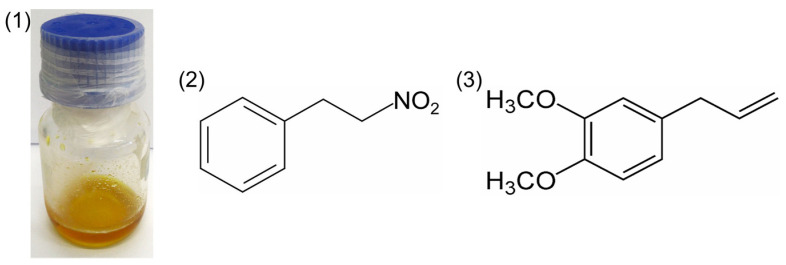
Essential oil of *Aniba canelilla* (1) and molecular structures of 1-nitro-2-phenylethane (2) and methyleugenol (3).

The constitution of the *A. canelilla* essential oil (Figure 1) was 1-nitro-2-phenylethane (86.637%), methyleugenol (12.700%) and benzaldehyde (0.663%). Meanwhile, isolated 1-nitro-2-phenylethane, the major compound of the essential oil, presented a content of 100%.

### 2.2. Determination of Minimum Inhibitory Concentration

The genera *Trichophyton* spp. and *Microsporum* spp. are known as etiologic agents involved in nail infections [20,21]. The susceptibility of strains of *Trichophyton rubrum* (TRU), *Trichophyton mentagrophytes* (TME) and *Microsporum canis* (MCA) to EOAC and NP were evaluated. In Table 1, the MIC for both EOAC and NP were compiled, compared to ciclopirox olamine. EOAC and NP inhibited the growth of the isolates tested with MICs ranging from 32 to 128 μg/mL and 27.7 to 110.8 μg/mL (TRU), respectively; 64 to 256 μg/mL and 27.7 to 221.7 μg/mL (TME), respectively; and 64 μg/mL and 27.7 μg/mL (MCA 38), respectively. For MCA 29, there was no growth inhibition at the concentrations tested (MIC > 256 μg/mL for EOAC and >221.7 μg/mL for NP).

### 2.3. Mechanism of Action of Aniba canelilla Essential Oil and Its Main Compound

The abilities of EOAC and NP to form a complex with the sterol of fungal membranes were evaluated by ergosterol binding assay. The MIC values of EOAC, NP and Amphotericin B—obtained by ergosterol binging assay against fungi strains *Trichophyton rubrum* 45, *Trichophyton rubrum* 51, *Trichophyton mentagrophytes* 40, *Trichophyton mentagrophytes* 60 and *Microsporum canis* 29 and *Microsporum canis* 38—are presented in Table 2.

Additionally, the antimicrobial effect on the integrity of the fungal cell wall was investigated by a sorbitol protection assay. The minimum inhibitory concentration for EOAC and NP and minimum effective concentration for Micafungin obtained by sorbitol protection assay before and after sorbitol addition against fungi strains *Trichophyton rubrum* 45, *Trichophyton rubrum* 51, *Trichophyton mentagrophytes* 40, *Trichophyton mentagrophytes* 60, *Microsporum canis* 29, and *Microsporum canis* 38 are presented in Table 3.

## 3. Discussion

The EOAC and NP antifungal susceptibilities were evaluated against filamentous fungi isolates of the genera *Trichophyton* spp. and *Microsporum* spp. to investigate its potential against onychomycosis. Previously performed studies have described the antifungal properties of EOAC against phytopathogens and *Candida* spp. [12,13,14,15]. The results observed in the present study confirm the antifungal activity of EOAC and NP against dermatophytes, being noticeable in eight out of nine clinical isolates (except for MCA 29), with inhibitory concentrations below 256 μg/mL. However, it is important to highlight that the susceptibility of a fungal species or genus causing a mycosis of medical importance may show variability in the inhibition of some strains, as the investigation of the expression of antifungal resistance has not been evaluated in these isolates. Normally, reduced susceptibility or even resistance to antifungal drugs is found in some strains of dermatophytes, although in tests, antifungal activity was noticeable against the strains for the compounds tested (EOAC and NP), as inhibitory action was evident in most of the isolates tested [22]. Additionally, ciclopirox olamine, a broad-spectrum fungicide for topical application, was used as positive control, exhibiting important activity at low concentrations (<2 μg/mL). It is important to note that ciclopirox olamine is, structurally, the N-oxide of a 2-hydroxypyridine derivative. On the other hand, 1-nitro-2-phenylethane, the main compound of EOAC, is an aromatic nitro compound formed through the biotransformation of phenylalanine [23,24]. The structural similarity between these substances with the presence of nitro groups may be an explanation for their antifungal activity.

It is important to highlight that as NP represents 86% of the essential oil and the EOAC and NP results were similar, it is suggested that 1-nitro-2-phenylethane plays an important role in the antifungal activity of *Aniba canelilla* essential oil, possibly being responsible for almost all its antifungal properties. Previous reports have suggested antifungal activity for 1-nitro-2-phenylethane from the investigation of minimum inhibitory concentration against clinical isolates of *Candida albicans*, *C. parapsilosis*, *C. tropicalis* and *Aspergilus fumigatus*, which varied from 170–1500 μg/mL [18]. These studies, added to the experimental findings, reinforce the antifungal properties of EOAC that can be attributed to NP.

However, it is noteworthy that antifungal properties against dermatophytes have also been reported for methyleugenol, which may also confer antifungal activity to the essential oil [25,26].

Assays designed to evaluate the mechanism of action of antifungal compounds mostly focus on the effect on the cell wall, the plasma membrane, since few molecules have any effect on the cell nucleus. In the present study, traditional assays were applied to evaluate the interaction with membrane ergosterol and cell wall components (using a sorbitol protection assay to evaluate the integrity of the cell wall) in addition to microscopic observation of the physiological alteration of the fungal reproduction structures of these selected isolates. Since the MIC of both EOAC and NP remained unchanged, it was suggested that both substances do not inhibit mechanisms that control the synthesis or existence of fungal cell wall. On the contrary, an increase in MIC was observed at all concentrations for Micafungin, a standard sorbitol protection substance capable of inhibiting mechanisms of cell wall synthesis and maintenance.

Additionally, the ability of EOAC and NP to form a complex with the sterol of fungal membranes were investigated by ergosterol binding assay. Despite the MIC of both EOAC and NP remaining unchanged, we proposed that the substances act by interacting with the fungal membrane ergosterol, since physiological alteration of the structures of fungal reproduction was noted in the selected isolates. In these observed changes, such as the modification of the shape of the conidial structures of the fungus, it was possible to observe that after exposure to the compound, there was permeability of the intracellular content due to the visually observed plasmolysis. Furthermore, a visible microscopic change in the presentation of the mycelium, with thinner and less germinated hyphae with conidia, allowed us to conclude that cytosolic extravasation through an osmotic process must be the main way of making the fungal culture susceptible during exposure to the tested compound [27]. No interaction studies were carried out with the nuclear material of the fungus, as we found possible interaction with the traditional targets investigated, and it is rare to observe an additive effect on the cell nucleus. For Amphotericin B (a standard substance used in the ergosterol assay, which interacts with fungal membrane ergosterol), an increase in MIC was observed at all concentrations.

A recent study has provided some insight into the mechanism of action of *Aniba canelilla* essential oil. Souza and coauthors [15] investigated the mechanism of action of EOAC against different phytopathogenic fungi (*Fusarium oxysporum*, *Fusarium solani*, *Aspergillus flavus*, *Aspergillus niger*, *Colletotrichum gloeosporioides*, *Colletotrichum musae*, *Colletotrichum guaranicola and Alternaria alternata*). They suggested that EOAC may induce changes in cell membrane permeability, causing alteration or disruption of the conidia cytoplasmatic membrane structure, inducing leakage of nucleic acids and proteins [15]. The authors also suggest that 1-nitro-2-phenylethane may play an important role in this mechanism of action, as the potential antifungal effect is generally ascribed to the main compounds present in the essential oil [15].

In this sense, the antifungal mechanism of action of EOAC and NP may be related to fungicide activity in the membrane cell of filamentous fungi. Further investigations regarding the mechanism of action of these substances should be proposed to clarify the inhibitory activity against dermatophytes.

## 4. Materials and Methods

### 4.1. Chemical Constitution of Aniba canelilla Essential Oil and Its Main Compound

The genetic material from Brazilian biodiversity used in this research was registered in the Sistema Nacional de Gestão do Patrimônio Genético e do Conhecimento Tradicional Associado (SisGen A0F4681 and ADF408C). After extraction of *Aniba canelilla* essential oil from barks collected at Mil Woods Itacoatiara Ltda. (Itacoatiara, Amazon State, Brazil), and after the isolation and purification of its main compound 1-nitro-2-phenylethane, both substances were characterized as previously described [10,16,28]. To extract *Aniba canelilla* essential oil, the barks of the plant were dried, grounded and subjected to hydrodistillation. After the extraction, the essential oil obtained was characterized by 5975C gas chromatography coupled with a 7890A mass spectrometry detector (GC-FID/MS, Agilent Technologies, Santa Clara, CA, USA), equipped with an automatic HS autosampler (CTC Analytics CombiPal, Basel, Switzerland). Briefly, 1-nitro-2-phenyletanhane, the *Aniba canelilla* essential oil major compound, was isolated and subsequently purified from the essential oil by two sequential low-pressure column chromatography systems with silica as a stationary phase and a gradient mobile phase composed of hexane–ethyl acetate. Finally, after extraction, 1-nitro-2-phenylethane was also analyzed by GC-FID/MS. EOAC and NP compounds were determined by their retention time and linear retention indices in addition to being identified by comparing retention indices and mass spectra using the NIST Mass Spectral Search Program 2.0 Database and the existing literature [29].

### 4.2. Fungal Strains

Nine clinical strains were used in this study, being *Trichophyton mentagrophytes* (TME 40, TME 60, TME 46, TME 1), *Trichophyton rubrum* (TRU 45, TRU 51, TRU 47) and *Microsporum canis* (MCA 29, MCA 38). All strains were deposited in the mycology collection of Laboratório de Micologia Aplicada at UFRGS (Porto Alegre, Brazil) and underwent confirmatory identification tests and characterization of their susceptibility to antifungals, thus allowing greater certainty in the description of the spectrum of action of the compounds being tested. The strains selected for tests were cultivated in Sabouraud agar medium with chloramphenicol in under pure conditions and subsequently inoculated into the antifungal susceptibility testing medium (RPMI Medium).

### 4.3. Antifungal Susceptibility Testing by Minimum Inhibitory Concentration

The minimum inhibitory concentrations (MICs) of *Aniba canelilla* essential oil and 1-nitro-2-phenylethane were determined by the broth microdilution method in accordance with the 38-A2 protocol for filamentous fungi (*Trichophyton* spp. and *Microsporum* spp.) [30]. Serial dilutions were made in RPMI 1640 medium, and experimental assays were carried out in triplicate (*n* = 3). The concentration ranges tested for EOAC and NP were, respectively, 256–0.5 μg/mL and 221.7–0.43 μg/mL. Ciclopirox olamine was used as the reference antifungal against dermatophyte fungi, and the concentration range tested was 0.5–0.00097 μg/mL. Cellular concentrations followed CLSI recommendations for both yeast and filamentous fungi for susceptibility tests by broth microdilution (with a turbidity of 0.5 McFarland). MICs were determined as the lowest concentration of compounds at which the microorganisms tested did not present visible growth after 96 h (TME 40, TME 60, TME 46, TME 1, TRU 45, TRU 51, TRU 47, MCA 29, and MCA 38). The sterility control (negative control medium free from drug) and positive control for fungal cell viability were performed in parallel. Optical microscopy was performed after the experimental assay in order to observe physiological alteration of the fungal reproduction structures.

### 4.4. Mechanism of Action

#### 4.4.1. Ergosterol Binding Assay

To evaluate the ability of the essential oil of *Aniba canelilla* and major compound 1-nitro-2-pheylethane to form a complex with the sterol of fungal membranes, an ergosterol assay was executed. The exogenous (qualitative) ergosterol determination technique was performed with and without the addition of ergosterol at concentrations of 100, 150, 200 and 250 μg/mL against *T. rubrum* (TRU 45 and TRU 51), *T. mentagrophytes* (TME 40 and TME 60) and *M. canis* (MCA 29 and MCA 38). Ergosterol was dissolved in dimethylformamide and the solution was added to RMPI-1640 medium (containing L-glutamine, without sodium bicarbonate, buffered to pH 7.0). The microplates were incubated at 35 °C for 96–192 h. The MICs were determined visually by the presence or absence of fungal growth. Amphotericin B was used as a control. Optical microscopy was performed after experimental assay to observe physiological alteration of the fungal reproduction structures. The assay was carried out in triplicate (*n* = 3).

#### 4.4.2. Sorbitol Protection Assay

The antimicrobial potential effect on the integrity of the fungal cell wall was investigated by a sorbitol protection assay. The MICs of the compounds were determined with and without addition of sorbitol (0.8 M) against the same strains above (Section 4.4.1). Sorbitol was dissolved in the RPMI-1640 culture medium (containing L-glutamine, without sodium bicarbonate, buffered at pH 7.0). The microplates were incubated at 35 °C and the MICs were determined visually by the presence or absence of fungal growth form the day 4 to the day 8 of incubation for the filamentous fungi. Micafungin was used as a control. Optical microscopy was performed after experimental assay to observe physiological alteration of the fungal reproduction structures. The assay was carried out in triplicate (*n* = 3).

## 5. Conclusions

*Aniba canelilla* essential oil and 1-nitro-2-phenylethane showed antifungal activity against dermatophytes, inhibiting the fungal growth in eight out of nine clinical isolates, with inhibitory concentrations below 256 μg/mL. Since 1-nitro-2-phenylethane is responsible for about 86% of the essential oil’s composition, it is suggested that this compound plays a prominent role in the antifungal activity of *Aniba canelilla* essential oil, possibly being responsible for almost all its antifungal properties. The mechanisms of action of EOAC and NP were also studied, and it is suggested that both compounds have fungicide effects on the fungal membrane, since the antifungal activity occurs through a modification of the shape of the conidial structures of the fungus, with permeability of the intracellular content due to visually observed plasmolysis and cytosolic extravasation occurring through an osmotic process. No activity related to interaction with the cell wall’s synthesis/formation was evidenced. These findings regarding the antifungal properties of *A. canelilla* essential oil and its main compound 1-nitro-2-phenylethane against dermatophytes are promising in view of the use of these substances as potential alternative plant-derived candidates for the treatment of ungual dermatophytosis.

## Figures and Tables

**Table 1:** Minimum inhibitory concentration (MIC, μg/mL) of the essential oil of *Aniba canelilla*, 1-nitro-2-phenylethane and ciclopirox olamine against dermatophytes (*n* = 3).

Dermatophytes (*n* = 9)	EOAC	NP	CPX
TME 60	256	221.7	2
TME 40	128	110.8	2
TME 1	256	221.7	1
TME 46	64	27.7	1
TRU 45	32	55.4	2
TRU 51	32	27.7	2
TRU 47	128	110.8	1
MCA 29	>256	>221.7	1
MCA 38	64	27.7	1

EOAC: essential oil of *Aniba canelilla*, NP: 1-nitro-2-phenylethane, CPX: ciclopirox olamine, TRU: *Trichophyton rubrum*, TME: *Trichophyton mentagrophytes*, MCA: *Microsporum canis*. The readings were performed at day 4.

**Table 2:** Minimum inhibitory concentration (μg/mL) obtained by ergosterol binding assay against six fungi isolates for the essential oil of *Aniba canelilla*, 1-nitro-2-phenylethane and Amphotericin B before and after adding ergosterol (*n* = 3).

Test Substance	Fungi Strains	MIC^1^	MIC^2^	MIC^3^	MIC^4^	MIC^5^
EOAC	TRU 45	64	64	128	128	32
	TRU 51	128	256	128	128	64
	TME 40	128	64	128	64	64
	TME 60	512	512	512	512	512
	MCA 29	512	1024	1024	512	1024
	MCA 38	128	64	64	64	64
NP	TRU 45	55.4	27.7	55.4	55.4	27.7
	TRU 51	110.88	110.88	55.4	55.4	27.7
	TME 40	221.7	55.4	55.4	55.4	55.4
	TME 60	443.5	443.5	>443.5	443.5	443.5
	MCA 29	443.5	443.5	443.5	443.5	443.5
	MCA 38	55.4	55.4	55.4	27.7	27.7
AFB	TRU 45	1	>8	>8	>8	8
	TRU 51	0.5	1	>8	>8	>8
	TME 40	0.5	1	2	2	1
	TME 60	1	1	8	8	8
	MCA 29	1	1	>8	>8	>8
	MCA 38	1	2	>8	>8	>8

MIC: minimum inhibitory concentration, EOAC: essential oil of *Aniba canelilla*, NP: 1-nitro-2-phenylethane, AFB: Amphotericin B, TRU: *Trichophyton rubrum*, TME: *Trichophyton mentagrophytes*, MCA: *Microsporum canis*. MIC^1^ corresponds to MIC without addition of commercial ergosterol. MIC^2^, MIC^3^, MIC^4^, and MIC^5^, correspond to MIC with addition of ergosterol at the concentration of 100, 150, 200, and 250 μg/mL, respectively. The readings were performed at day 8.

**Table 3:** Minimum inhibitory concentration (μg/mL) for the essential oil of *Aniba canelilla* and 1-nitro-2-phenylethane, and minimum effective concentration (μg/mL) for Micafungin obtained by sorbitol protection assay before and after sorbitol addition against six fungi isolates.

Fungi Strains	Readings	EOAC (MIC)		NP (MIC)		MCF (MEC)	
		−/Sorbitol	+/Sorbitol	−/Sorbitol	+/Sorbitol	−/Sorbitol	+/Sorbitol
TRU 45	Day 4	64	32	110.88	110.88	-	-
	Day 8	64	32	221.7	110.88	0.015	0.250
TRU 51	Day 4	128	32	110.88	55.4	0.0625	0.031
	Day 8	128	64	110.88	55.4	-	-
TME 40	Day 4	128	32	55.4	27.7	0.031	0.031
	Day 8	256	128	55.4	27.7	-	-
TME 60	Day 4	>512	>512	443.5	443.5	0.015	0.250
	Day 8	>512	>512	>443.5	>443.5	-	-
MCA 29	Day 4	512	512	443.5	443.5	0.031	0.5
	Day 8	>512	256	>443.5	>443.5	0.125	0.5
MCA 38	Day 4	64	32	110.88	55.4	-	-
	Day 8	64	32	110.88	55.4	0.5	>0.5

MIC: minimum inhibitory concentration, MEC: minimum effective concentration, EOAC: essential oil of *Aniba canelilla*, NP: 1-nitro-2-phenylethane, MCF: Micafungin, TRU: *Trichophyton rubrum*, TME: *Trichophyton mentagrophytes*, MCA: *Microsporum canis*.

## Data Availability

The authors declare that the data from the study are protected for privacy reasons.
